# Embryonic toxico-pathological effects of meglumine antimoniate using a chick embryo model

**DOI:** 10.1371/journal.pone.0196424

**Published:** 2018-05-25

**Authors:** Ahmad Khosravi, Iraj Sharifi, Hadi Tavakkoli, Amin Derakhshanfar, Ali Reza Keyhani, Zohreh Salari, Seyedeh Saedeh Mosallanejad, Mehdi Bamorovat

**Affiliations:** 1 Leishmaniasis Research Center, Kerman University of Medical Sciences, Kerman, Iran; 2 Department of Clinical Science, School of Veterinary Medicine, Shahid Bahonar University of Kerman, Kerman, Iran; 3 Center of Comparative and Experimental Medicine, Shiraz University of Medical Sciences, Shiraz, Iran; 4 Obstetrics and Gynecology Center, Afzalipour School of Medicine, Kerman University of Medical Sciences, Kerman, Iran; Gaziosmanpasa University, TURKEY

## Abstract

Leishmaniasis is one of the diverse and neglected tropical diseases. Embryo-toxicity of drugs has always been a major concern. Chick embryo is a preclinical model relevant in the assessment of adverse effects of drugs. The current study aimed to assess embryonic histopathological disorders and amniotic fluid biochemical changes following meglumine antimoniate treatment. The alteration of vascular branching pattern in the chick’s extra-embryonic membrane and exploration of molecular cues to early embryonic vasculogenesis and angiogenesis were also quantified. Embryonated chicken eggs were treated with 75 or 150 mg/kg of meglumine antimoniate. Embryo malformations, growth retardation and haemorrhages on the external body surfaces were accompanied by histopathological lesions in the brain, kidney, liver and heart in a dose-dependent manner. Significant rise occurred in the biochemical indices of alkaline phosphatase, aspartate aminotransferase, alanine aminotransferase and amylase in the amniotic fluid. Quantification of the extra-embryonic membrane vasculature showed that the anti-angiogenic and anti-vasculogenic effects of the drug were revealed by a significant decrease in fractal dimension value and mean capillary area. The relative expression levels of vascular endothelial growth factor A and vascular endothelial growth factor receptor 2 mRNA also significantly reduced. Concerns of a probable teratogenicity of meglumine antimoniate were established by data presented in this study. It is concluded that tissue lesions, amniotic fluid disturbance, altered early extra-embryonic vascular development and gene expression as well as the consecutive cascade of events, might eventually lead to developmental defects in embryo following meglumine antimoniate treatment. Therefore, the use of meglumine antimoniate during pregnancy should be considered as potentially embryo-toxic. Hence, physicians should be aware of such teratogenic effects and limit the use of this drug during the growing period of the fetus, particularly in rural communities. Further pharmaceutical investigations are crucial for planning future strategies.

## Introduction

Leishmaniasis is a major medical and veterinary problem worldwide, affecting 12 million people in 98 countries with two million new cases each year and 350 million people at risk. Presently, the disease is the third most important parasitic disease (after malaria and African trypanosomiasis) and remains among the 9 important infectious diseases in terms of global impact, and accounts for over 20,000–40,000 deaths per annum and 2.4 Disability Adjusted Life Years lost [1]. Several clinical and epidemiological forms are predominant, depending on the causative species. Currently, 22 species are responsible for various clinical forms including cutaneous, mucocutaneous, visceral, diffuse and post kala-azar dermal leishmaniasis in five continents. Most of the cases are largely hidden, concentrated in remote rural areas, and are more common in urban slums and shanty towns. The disease is also largely salient, as the people affected have little political voice with low standard of living in terms of socio- economic and hygienic conditions[2].

Meglumine antimoniate (MA) is a pentavalent antimony compound considered as a first-line agent in the treatment of leishmaniasis. Several mechanisms of action of pentavalent antimony have been suggested: ‘‘Prodrug Model”, ‘‘Intrinsic Antileishmanial Activity Model” and ‘‘Immune Activation Model”[3]. The current commercial MA drug is Glucantime^®^, produced by Sanofi-Aventis, France. It is well established that following injection, MA rapidly crosses the placenta into the fetal circulation[4]. Furthermore, the drug is categorized in group C of the United States Food and Drug Administration (FDA) pharmacological drug and inadequate research has been done to determine its adverse effects during pregnancy[5].

The normal intra-uterine development of the fetus has always been a major concern. Some of the most important factors that influence this biological phenomenon are the toxic effects of drugs and the processes governing the genesis and proliferation of the vascular network. Consumption of certain drugs during pregnancy is related with an increased risk of gestational abnormalities, including congenital malformations, histopathological injuries, growth retardation and alteration in the biochemical characteristics of amniotic fluid[6,7].

Drugs may also possess vasculo-toxic properties, causing cell damage and affecting the embryonic peripheral vascular development. These activities have been documented in various experimental investigation [8]. Formation and proliferation of the vascular network are known as vasculogenesis and angiogenesis and are the critical steps associated in embryo growth. Vasculogenesis is the *de novo* generation of vessels from angioblasts, whilst angiogenesis is concerned with the branching and sprouting of new vasculature from pre-existing ones. Several pathways, growth factors and proteins are critical for the regulation, promotion and inhibition of these processes. The most important protein in the foregoing processes is the Vascular Endothelial Growth Factor A (VEGF-A) which is generated by mesenchymal organs, and attaches to several subfamilies of tyrosine kinase receptors such as VEGF-R1, -R2 and -R3[9].

Determination of the adverse effects of chemical elements and drugs needs the use of a preclinical model. The chick embryo provides a suitable model for *in vivo* evaluation of the toxicity, biocompatibility, biodistribution and pharmacokinetics of drug due to simplicity, low cost, good reproducibility of results, reduced ethical and legal aspects, and the mother does not influence the pharmacokinetics of the drug [10–12]. Furthermore, the chick’s extra-embryonic membrane (EEM) is developed to assess the effects of angiogenic/anti-angiogenic and vasculogenic/anti-vasculogenic factors and compounds[13–15]. The vascular plexus of the chick’s EEM is simple and contains undifferentiated vessels which allow it to branch progressively during embryonic growth. In this regard, it may produce congenital toxicity and cause adverse effect on the development of the human fetus during pregnancy. Although increasing use and production of MA are predicted in some regions of the globe [16], little has been reported on the toxic and pathological effects of this compound on embryonic development and vasculature. In addition, the exact mechanisms by which it affects vascular genesis and expansion are not yet fully understood.

The present investigation aimed to reply the following questions:

(i) Does MA cause adverse and pathological effects on the embryo?
(ii) Does MA alter the biochemical parameters of the amniotic fluid following treatment?
(iii) Does MA alter the development of the EEM-vasculature?
(iv) Does MA alter the expression of VEGF-A and its receptor in the extra-embryonic vasculature?

To answer these questions, a chick embryo model was used. Computerized programs were also applied to quantify the branching pattern and morphometric evaluation of vessel density in the chick’s EEM in order to demonstrate the MA-induced anti-angiogenic/anti-vasculogenic activities. Finally, the results were joint with real-time PCR data to assess the effect of drug on molecular expression, which is associated with vascular genesis.

## Materials and methods

This study is presented in 3 experiments:

Experiment 1, where chicken eggs were treated with MA at dosages of 75 and 150 mg per kg egg-weight at day 4 of incubation. The parameter studied included embryonic weights, gross and histological alterations.

Experiment 2, chicken eggs were treated with MA at dosages of 75 and 150 mg per kg egg-weight and parameters studied included amniotic fluid biochemistry.

Experiment 3, where chicken eggs were treated with MA at 24, 48 and 72 h of incubation, at dosages of 75 and 150 mg per kg egg-weight. Parameters studied included all those related to test early embryonic vascular development and angiogenesis.

### Hatching eggs

Fertile chicken eggs (Ross 308) with the average egg-weight of 54.4 ± 0.8 g were provided from the Mahan Breeder Company, Kerman, Iran. In this company, the breeder birds are reared under standard condition to attain optimum bird performance.

### Drug

Glucantime^®^ injectable solution was obtained from Sanofi-Aventis Pharmaceuticals Ltd., Paris, France. Each milliliter of drug contains 300 mg MA.

### Experiment 1

#### Histopathological lesions of MA in the chick embryo

Various dosages of MA were used to evaluate histopathological lesions of the drug in a chick embryo model. The assays are described as follows.

#### Embryo treatment

Fertilized eggs were incubated in an electrical incubator (Belderchin Damavand Co. PLC-DQSH, Tehran, Iran) at 37.5°C and 60% relative humidity. Then, they were divided into three equal treatment groups of 30 eggs each. Group 1: phosphate buffered saline injected group (control group) in which embryonated eggs were injected with sterile phosphate buffered saline of 0.5 ml/egg on day 4 of the incubation period, to Hamburger–Hamilton developmental stage 22–24 [17]. The eggs of groups 2 and 3 were likewise treated with MA at dosages of 75 or 150 mg per kg egg-weight, respectively. For preparation of the mentioned dosages, the MA was dissolved in PBS and the constant volume of 0.5 ml/egg (PBS+MA) was injected. Embryos were treated by direct inoculation into the yolk sac and allowed to develop for further days [17].

#### Gross evaluation and measurement

The embryos were examined at 3 different time intervals: days 6, 12 and 18 of the incubation period. At the end of each time point, the embryos (n = 30) were humanely killed by cooling [18]. The eggs were opened at the blunt end and embryos were removed to study any gross injuries on the body surface. The average embryo-weight/egg-weight in grams of each group was computed. Body weight was measured by a digital scale (Sartorius TE 1535, Germany, with a range up to 150 g reading to ± 0.001 g).

#### Histopathological evaluation

The tissues including brain, liver, kidney and heart were sampled and fixed in 10% neutral buffered formalin. Serial sections of paraffin embedded tissues of 5 μm thickness were cut by a microtome (Slee-Germany) and stained with hemotoxylin and eosin (H&E) for investigating under a light microscope.

### Experiment 2

#### Effect of MA on the biochemical parameters of the amniotic fluid sampling

Embryo treatment was similar to that described in the previous section. The amniotic fluid was sampled on day 14 of the incubation period [19]. After removing the egg shell and shell membranes at the blunt end of the egg, the allantoic membrane and fluid were obtained to facilitate the amniotic fluid sampling. The amniotic fluid was collected by an 18 gauge syringe (n = 12 per treated group). The samples were centrifuged at 3000 g for 10 min and then supernatant was taken for biochemical analysis. Enzymatic activities of alkaline phosphatase (ALP), aspartate aminotransferase (AST), alanine aminotransferase (ALT) and amylase were determined. The levels of urea, creatinine (CT) and glucose were also quantified using the commercial kits (Parsazma Company, Iran, Tehran).

### Experiment 3

#### Effect of MA on the embryonic vasculogenesis and angiogenesis

The effect of MA on early embryonic vasculogenesis and angiogenesis was determined by analysis of vascular branching pattern and morphometric evaluation of capillary density from the chick’s EEM. The details are presented as below.

#### Embryo treatment and image acquisition

Ross 308 fertilized eggs were purchased and incubated. The incubation condition was similar to that described in the previous section. A pinpoint hole of approximately 0.5 mm was drilled in the eggshell and the outer shell membrane at the blunt end after 24 h of incubation. The eggs were treated with 50 μl of either MA or sterile phosphate buffered saline as sham control. They were re-treated at 2 different time points: 24 and 48 h following the first treatment (e.g., 48 and 72 h total developmental stage, respectively). The drug was applied in a single drop on the inner shell membrane, using a 50 μl Hamilton syringe. This method of treatment has already been described [20]. After each treatment, the exposed hole was sealed with warmed paraffin (Merck, Darmstadt, Germany) and the eggs were placed back into the incubator under the mentioned condition. On day 4 of incubation, live embryos were selected and those that died during the incubation period (e.g., due to the effect of drug on the blastodisc and vascular plexus or manipulation) were excluded from the study. Finally, treated embryos were assigned to three groups as follows: group 1 (n = 10): phosphate buffered saline treated group (untreated control group), groups 2 (n = 10) and 3 (n = 10): MA-treated groups, in which, embryonated eggs were treated with MA at dosages of 75 or 150 mg per kg egg-weight, respectively. For preparation of the mentioned dosages, the MA was dissolved in PBS and the constant volume of 50 μl/egg (PBS+MA) was injected. A window of 25 mm by 25 mm was made in the eggshell, to allow microscopic imaging of the EEM-vasculature. High resolution images (4000 × 3000 pixels) were captured using a stereomicroscope (Luxeo 4D Stereozoom Microscope, Labomed, CA, USA) attached to Canon SX200 camera supported by Luxeo software. Images were obtained from the EEM of the embryos and their area vasculosa. The captured images were saved as *.tif files using a 14.5 inch PC (Intel Core i3-390M, 2.66 GHz).

#### Vascular branching pattern analysis

Computerized analysis was made using the image analyzer softwares MATLAB^®^ (Mathworks Matlab R2015a), ImageJ^®^ 1.48 (National Institutes of Health, Bethesda, Maryland, USA) and Digimizer^®^4.3.0 (MedCalc Software, Mariakerke, Belgium). A certain area was extracted from the captured images. The extracted area, approximately 177 mm^2^ containing 1100×1800 pixels, was identified at the right-lateral vitelline vascular plexus. The images were converted into 8-bit format and processed to increase edge detection for later steps. Finally, their color level was reduced to binarized (black and white) format and transformed into skeletonized pictures. Skeletonized picture presents the structural shape of the object. The vascular branching pattern was specifically estimated for changes in fractal dimension (Df) value. The Df was planned using the box counting method on the processed images as previously described elsewhere[20].

#### Morphometric evaluation of capillary density

A certain area within the right-lateral vitelline vascular plexus was designated and the contrast was enhanced. Efforts were made to select the constant areas in order to avoid subjectivity in image analysis. The selections were reduced to a binarized format. From these, areas without any branch vessel were selected for evaluation. Five of such areas per case were identified and the percentage of the areas containing black pixels was calculated. The black pixels of the images indicate the red color or blood in the main images. The mean of all areas quantified in each image is expressed as the mean capillary area (MCA) [21].

#### Effect of MA on the of VEGF-A and VEGF-R2 expression

Relative expression levels of VEGF-A and VEGF-R2 genes were assessed by quantitative real-time PCR (qPCR) assay. Briefly, RNA was extracted from the chick’s EEM by the RNeasy^®^ mini kit (Qiagen, Chatsworth, CA) according to the manufacturer's protocol (n = 4 per treated group). RNA concentration (ng) and purity (260 nm: 280 nm) were assessed spectrophotometrically using the NanoDrop ND-1000 (NanoDrop ND-1000, Thermo Scientific, Wilmington, DE, USA). cDNA was synthesized by TaKaRa Prime Script™ RT reagent kits (Takara Bio, Inc., Shiga, Japan) and reverse transcription was carried out on 500 ng of the total RNA at 37°C for 15 min. Real-time quantitative PCR (qPCR) reaction was performed in duplicate with the Rotorgene Cycler system (Rotorgene 3000 cycler system (Corbett Research, Sydney, Australia) using a SYBR Green assay (SYBR Premix Ex Taq™ II, Takara Bio, Inc., Shiga, Japan) according to the recommended protocol. The specific primers and reference gene (B2M and GAPDH) sequences are listed in Table 1 [20]. The primers amplified an 86 bp fragment in the VEGF-A and VEGF-R2 mRNA genes. At first, a holding treatment at 95°C for 1 min was performed and then amplification was done for 40 cycles (10 s at 95°C for denaturation of DNA, 15 s at 60°C for primer annealing and 20s at 72°C for extension). Expression levels were calculated relative to the expression levels of the selected reference gene.

**Table 1:** The specific primers and reference gene sequences for real-time quantitative PCR.

Gene (*Gallus gallus)*	Forward sequence (5′–3′)	Reverse sequence (5′–3′)	Product size (bp)
**VEGF-A**	CAATTGAGACCCTGGTGGAC	TCTCATCAGAGGCACACAGG	86
**VEGF-R2**	GCCAACTCTATGGCAGAAGC	CTGAACACCATGCCACTGTC	86
**B2M**	GTGCTGGTGACCCTGGTG	CAGTTGAGGACGTTCTTGGTG	113
**GAPDH**	CCTCTCTGGCAAAGTCCAAG	GGTCACGCTCCTGGAAGATA	176

### Ethics statement

The study was performed according to the suggested European Ethical Guidelines for the care of animals in experimental investigations, in line with guidelines of Kerman University of Medical Sciences and was approved (project number 94/974/205, Ethic number IR.KMU.REC.1394.525) by the Animal Ethics Committee of the Research Council of the Kerman University of Medical Sciences, Iran.

### Statistical analysis

Statistical analysis was performed by SPSS version 20 (SPSS Inc., Chicago, IL, USA). The Fisher's exact and repeated measurement tests were used to determine the significant differences in lesion occurrence and the embryo weight between experimental groups, respectively. One-way analysis of variance was applied followed by Tukey's test to assess the significance of differences in the biochemical parameters of the amniotic fluid: Df value, MCA and gene expression. A p-value of <0.05 was defined statistically significant.

## Results

### Gross evaluation of the external body features

**Fig 1:**
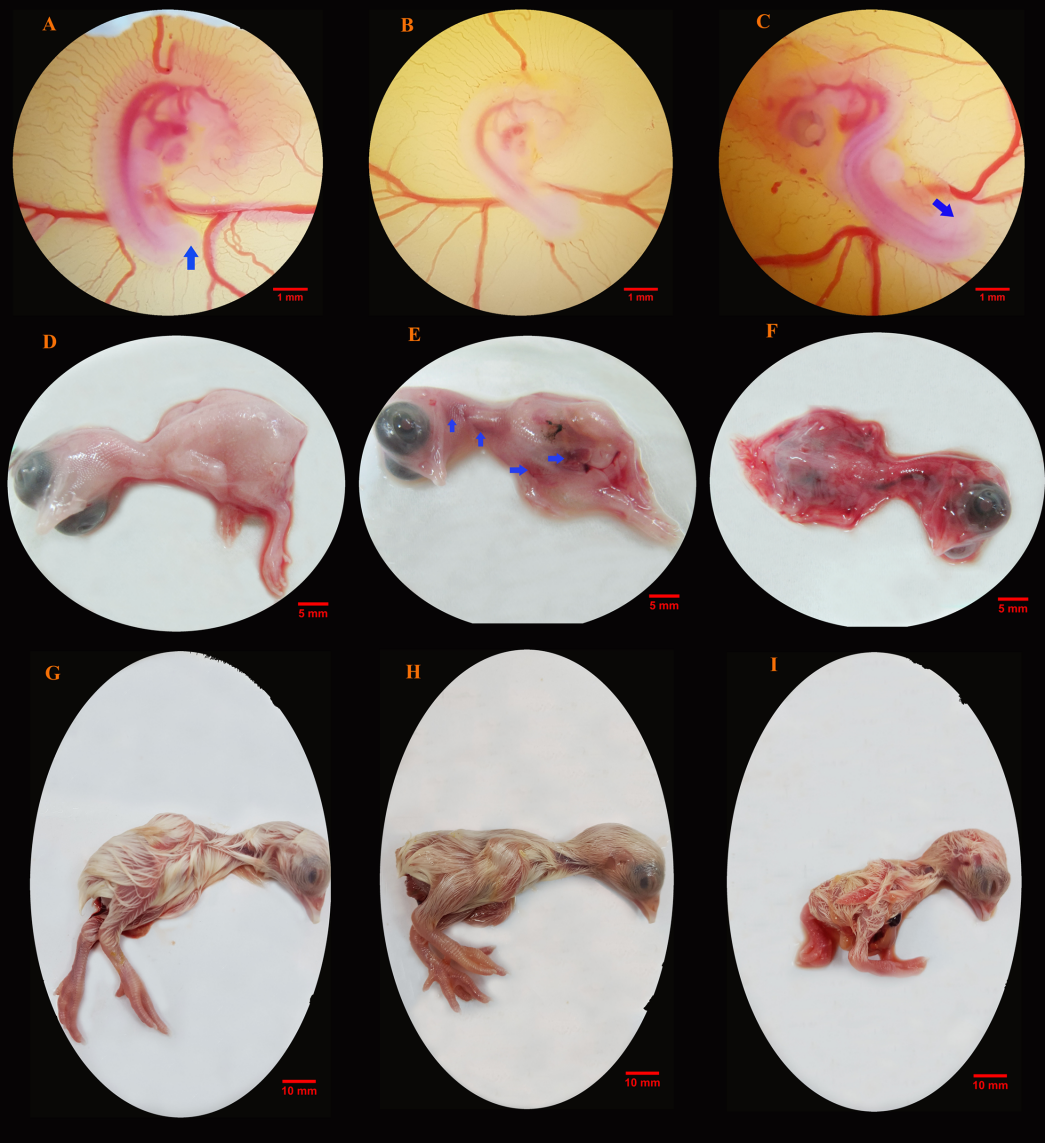
Gross lesions by meglumine antimoniate in the chick embryo model. Lesions during the first (A-C), second (D-F) and third (G-I) trimesters of the growing period are presented with comparison between the control (A, D and G) and meglumine antimoniate-treated embryos at dosages of 75 mg (B, E and H) or 150 mg (C, F and I) per kg egg-weight. Embryos with normal C-shape at the craniocaudal body axis; the lower limbs and tail tip grow ventrally in the same direction (arrow) (A and B). Twisting and lordosis in the lumbosacral region of body which made the embryo to be S-shaped at the craniocaudal axis; the lower limbs grow dorsally to the tail tip (arrow) (C). Normal view of the external body surfaces is obtained (D). Local (arrows) (E) and general congestion (F) are noticed on the skin. Normal embryos with no gross disorder are seen (G and H). Moderate general congestion and growth retardation are observed (I).

Gross lesions by MA in the chick embryo model during the first, second and third trimesters of the growing period (days 6, 12 and 18 of the incubation period, respectively) are described in Fig 1. Embryos treated with 150 mg per kg egg-weight of MA, demonstrated malformations (20%; n = 6) in the first trimester. The affected embryos had twisting and lordosis in the lumbosacral region of the body, which made them S-shaped at the craniocaudal axis. The control and other embryos showed a normal C-shape of the longitudinal body axis. Furthermore, in the former embryos (exposed to 150 mg of MA), the lower limbs grew dorsally to the tail tip, whereas in the normal situation, the lower limbs and tail tip grew ventrally in the same direction (Fig 1A–1C).

In the second trimester, the main symptom manifested as congestion on the external body surfaces. The lesion, generally noticed as a local (Fig 1E) or general (Fig 1F) congestion was located on the skin. However, the worst condition was seen in embryos treated with high dosage of MA.

In the third trimester, in 36.7% (n = 11) of embryos that were treated with 150 mg per kg egg-weight of MA, the drug caused disorders characterized by moderate general congestion and growth retardation, while all the control embryos and those that received low dose of the drug, exhibited no gross disorder (Fig 1G–1I) (S1 Fig).

### Embryo weight

**Fig 2:**
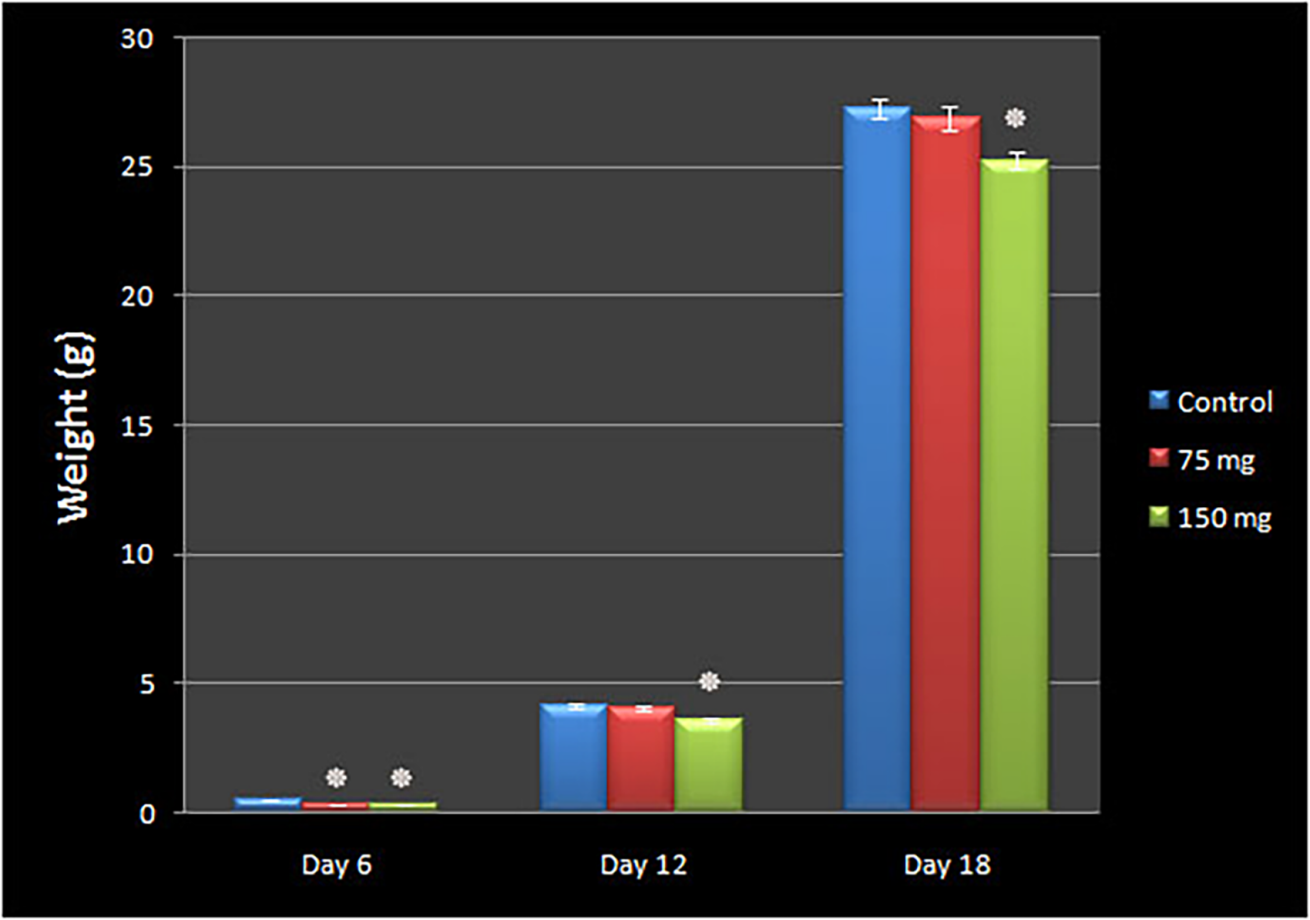
The weight of the chick embryos following meglumine antimoniate treatment. The weight at days 6, 12 and 18 of the incubation period is presented with comparison between control (n = 30) and meglumine antimoniate-treated embryos at dosages of 75 (n = 30) or 150 (n = 30) mg per kg egg-weight. The embryos that received 150 mg meglumine antimoniate per kg egg-weight were significantly lower than the controls at the above maintained time points. The embryos which received 75 mg per kg egg-weight of meglumine antimoniate, showed a weight loss comparable to the controls only at day 6 of the incubation period (*p<0.01, repeated measurement tests).

The weight of the treated embryos at three different time points (days 6, 12 and 18 of the incubation period) are demonstrated in Fig 2. The MA-treated embryos that received the high dosage of drug (150 mg per kg egg-weigh) were lighter than the control at the above maintained time points (0.29 ± 0.01 g (95% CI 0.26–0.32), p < 0.001, on day 6; 3.56 ± 0.09 g (95% CI 3.37–3.75), p < 0.001, on day 12; 25.24 ± 0.32 g (95% CI 24.56–25.91), p = 0.002, on day 18).

The embryos that received 75 mg per kg egg-weight of MA showed a weight loss comparable to the control only at day 6 of the incubation period (0.33 ± 0.01 g (95% CI 0.30–0.35), p < 0.001 on day 6; 4.03 ± 0.09 g (95% CI 3.83–4.24), p = 0.673, on day 12; 26.92 ± 0.46 g (95% CI 25.97–27.86), p = 0.843, on day 18). The control embryos had normal weight (0.49 ± 0.01 g (95% CI 0.46–0.53) on day 6; 4.15 ± 0.08 g (95% CI 3.97–4.32) on day 12; 27.23 ± 0.38 g (95% CI 26.45–28.00) on day 18 (S1 Table).

### Histopathological lesions

**Fig 3:**
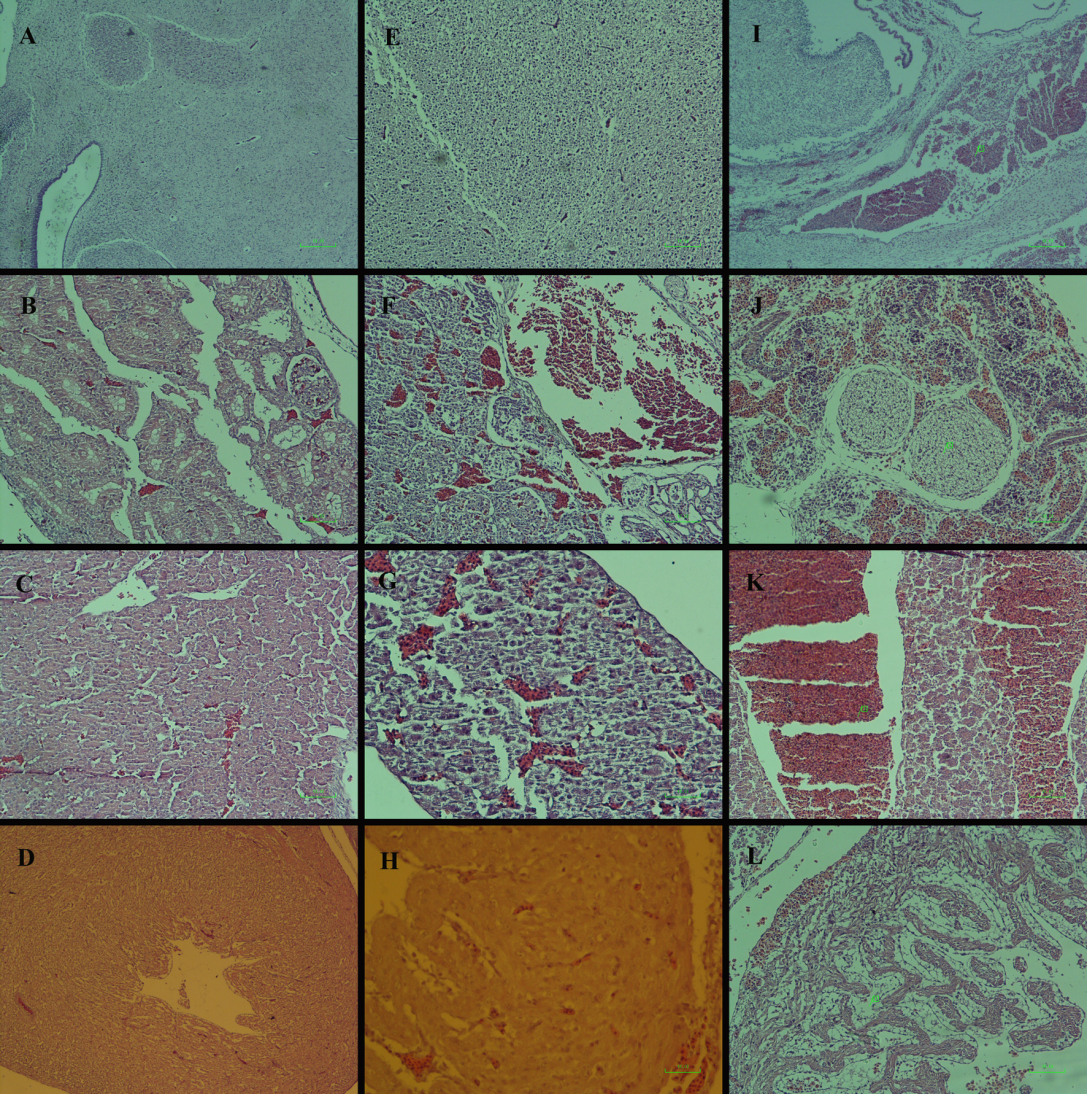
Histopathological lesions by meglumine antimoniate in the chick embryo model. Lesions were observed on day 12 of the incubation period (8 days after treatment) with comparison between the control (A-D) and meglumine antimoniate-treated embryos at dosages of 75 (E-H) or 150 (I-L) mg per kg egg-weight. Photomicrographs were obtained from the various tissues, including brain (A-I), kidney (B-J), liver (C-K) and heart (D-L). Normal structure of the brain, kidney, liver and heart are seen, respectively (A, B, C and D). The brain is normal with no lesion (E). Severe hyperemia is seen in the kidney and liver (F and G). Hyperemia and sign of degeneration characterized by lack of striation and hyalinization of cytoplasm are seen in the heart (H). Dilation and hyperemia are noticed in the meninges (I). The kidney is hyperemic with large and deformed glomeruli (J). The liver is characterized by severe hyperemia and severe dilation of central veins (K). Cardiomyocytes are not developed and replaced with myxomatous tissue (L). H&E staining.

Histopathological lesions after MA administration on chick embryos were detected in the brain, kidney, liver and heart. At day 12 of the incubation period (8 days after treatment with 75 mg per kg egg-weight of MA), the brain of the embryos was normal, while severe hyperemia was noted in the kidney and liver (Fig 3E–3G). In the heart, hyperemia and signs of degeneration characterized by lack of striation and hyalinization of cytoplasm were observed (Fig 3H).

At dosage of 150 mg per kg egg-weight of MA, dilation and hyperemia were seen in the meninges (Fig 3I). The kidney was hyperemic with large and deformed glomeruli (Fig 3J). The livers showed severe hyperemia and severe dilation of central veins (Fig 3K). In the heart, cardiomyocytes did not develop and were replaced with myxomatous tissue (Fig 3L).

**Fig 4:**
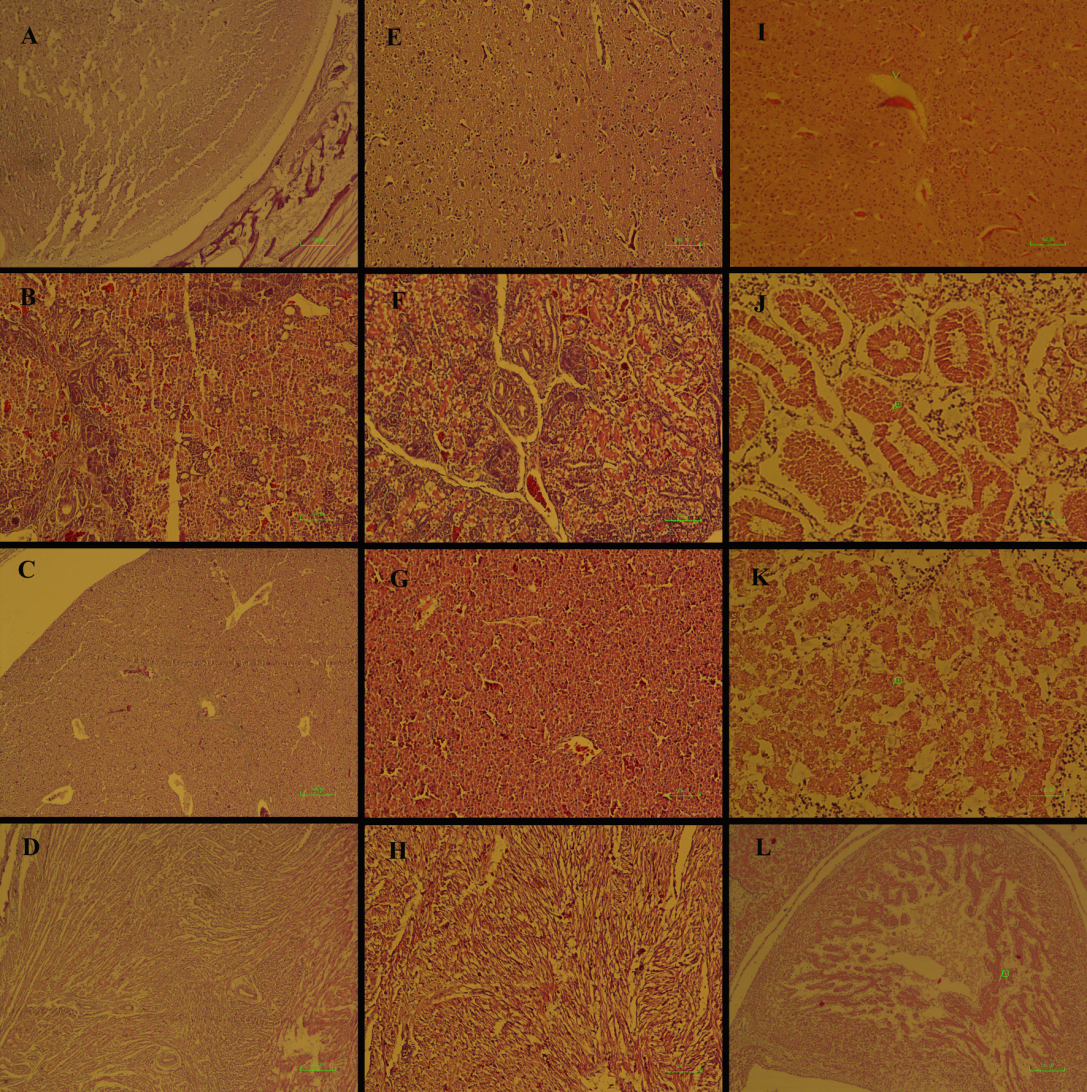
Histopathological lesions by meglumine antimoniate in the chick embryo model. Lesions were observed on day 18 of the incubation period (14 days after treatment) with comparison between the control (A-D) and meglumine antimoniate-treated embryos at dosages of 75 (E-H) or 150 (I-L) mg per kg egg-weight. Photomicrographs were obtained from the various tissues including brain (A-I), kidney (B-J), liver (C-K) and heart (D-L). Normal structure of the brain, kidney, liver and heart are seen, respectively (A, B, C and D). The structure of the brain, kidney and heart is normal (E-G). Mild hyperemia is evident in the liver (H). Hyperemia, edema and dilation of Virchow-Rabin space are seen in the brain (I). Necrosis of the renal tubules is seen (J). Hepato-cellular degeneration is observed (K). Cardiac myocytes are deformed and under-developed (L). H&E staining.

On day 18 of the incubation period (14 days after treatment with 75 mg per kg egg-weight of MA), the structure of the brain, kidney and heart was normal, although mild hyperemia was evident in the liver (Fig 4E–4H). At dosage of 150 mg per kg egg-weight of MA, hyperemia, edema and dilation of Virchow-Rabin space were seen in the brain (Fig 4I). Necrosis of the renal tubules and hepato-cellular degeneration occurred (Fig 4J and 4K). Besides, cardiac myocytes were deformed and under-developed (Fig 4L) (S2 Fig).

### Biochemical parameters of the amniotic fluid

Table 2 shows the biochemical parameters of the chick embryo’s amniotic fluid in the control and treated embryos. Administration of MA revealed changes in some parameters. The levels of ALP, AST, ALT and amylase were increased in the MA-treated embryos as compared to the control (p<0.01), while no significant differences were seen in the level of urea, CT and glucose. There was no significant difference between embryos that received different dosages of the drug (S2 Table).

**Table 2:** Biochemical parameters of amniotic fluid in chick embryo following meglumine antimoniate treatment (data are acquired on the 14th day of the incubation period).

Biochemical parameters	Control	Meglumine antimoniate			
		(75 mg per kg egg-weight)	P-value	(150 mg per kg egg-weight)	P-value
**ALP (IU/L)**	6.61 (2.97–10.25)	269.83 (109.41–430.25)	0.004	299 (164.44–433.55)	0.001
**AST (IU/L)**	8.69 (4.22–13.15)	178.16 (75.99–280.33)	0.006	200.33(100.18–300.48)	0.002
**ALT (IU/L)**	2.23 (1.79–2.66)	59.41 (32.12–86.71)	0.000	56.5 (30.85–82.14)	0.001
**Amylase (mg/L)**	5.07 (3.48–6.66)	50.16 (5.88–94.45)	0.113	79.75 (37.84–121.65)	0.005
**Urea (mg/dL)**	9.76 (7.41–12.11)	17.50 (6.71–28.28)	0.147	6 (4.39–7.6)	0.620
**CT (mg/dL)**	0.45 (0.38–0.52)	0.45 (0.32–0.57)	0.997	0.35 (0.30–0.41)	0.221
**Glucose (mg/dL)**	24.92 (5.14–44.7)	17.91 (8.40–27.42)	0.711	15.58 (8.62–22.54)	0.548

Values are mean and 95% confidence interval for mean, one-way ANOVA. ALP, alkaline phosphatase; AST, aspartate aminotransferase; ALT, alanine aminotransferase; CT, creatinine.

### Vascular branching pattern and Df value

**Fig 5:**
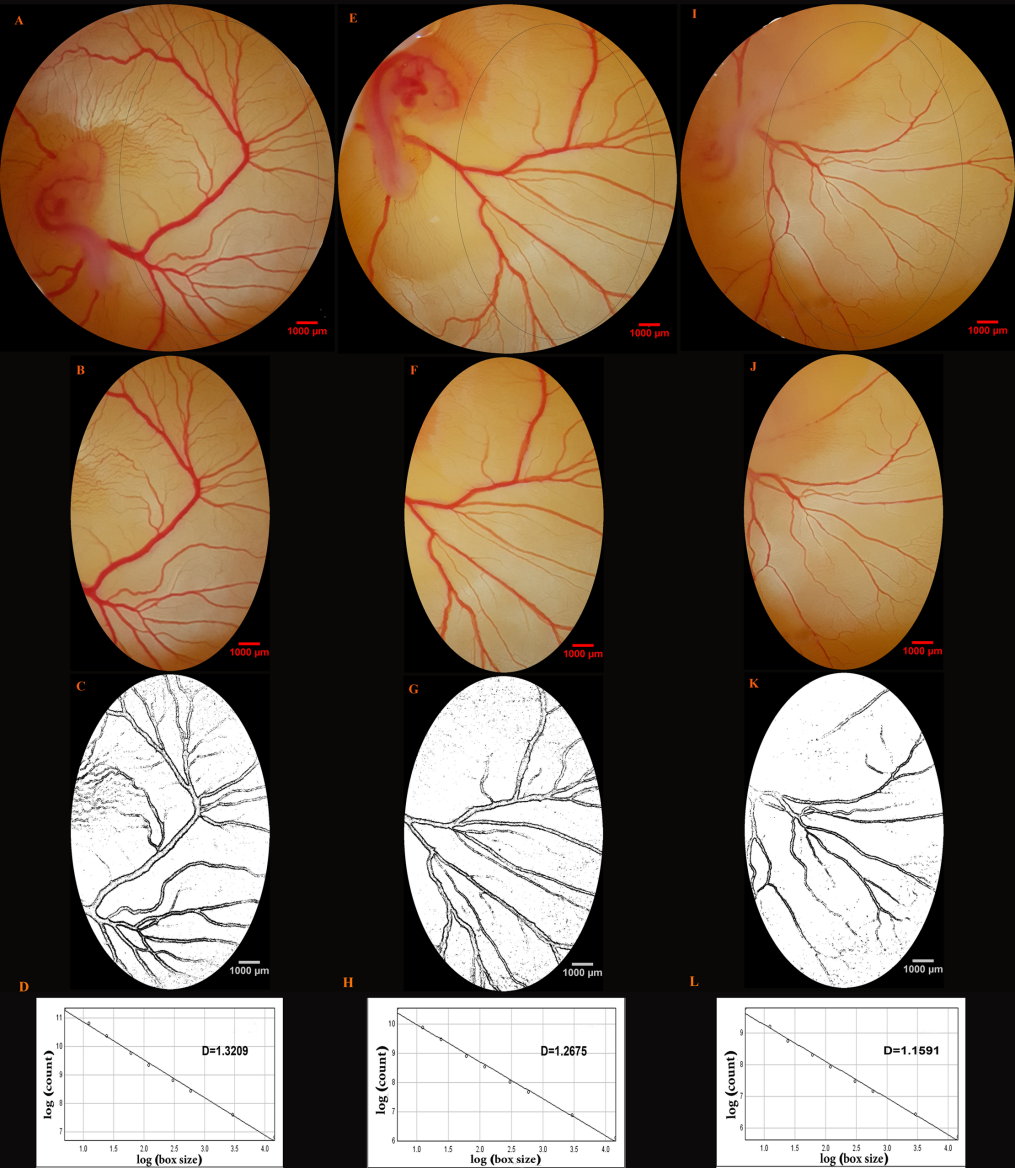
Generation of the fractal dimension (Df) value from the captured images. The day 4 embryos and their area vasculosa are presented to illustrate the image manipulations required to generate the Df value. The images were captured from the embryo of the control (A-D) and meglumine antimoniate at dosages of 75 (E-H) or 150 (I-L) mg per kg egg-weight. The oval is located right before the first main branch of the right lateral vitelline vein, and has included a size of 177 mm^2^ (A, E and I). The extracted areas were converted into 8-bit format and processed to increase edge detection (B-C, F-G and J-K). The Df is measured from the slope of the regression line using the box counting method (D, H and L).

The generating steps of the fractal dimension (Df) value from the captured images are presented in Fig 5 (S3 Table).

**Fig 6:**
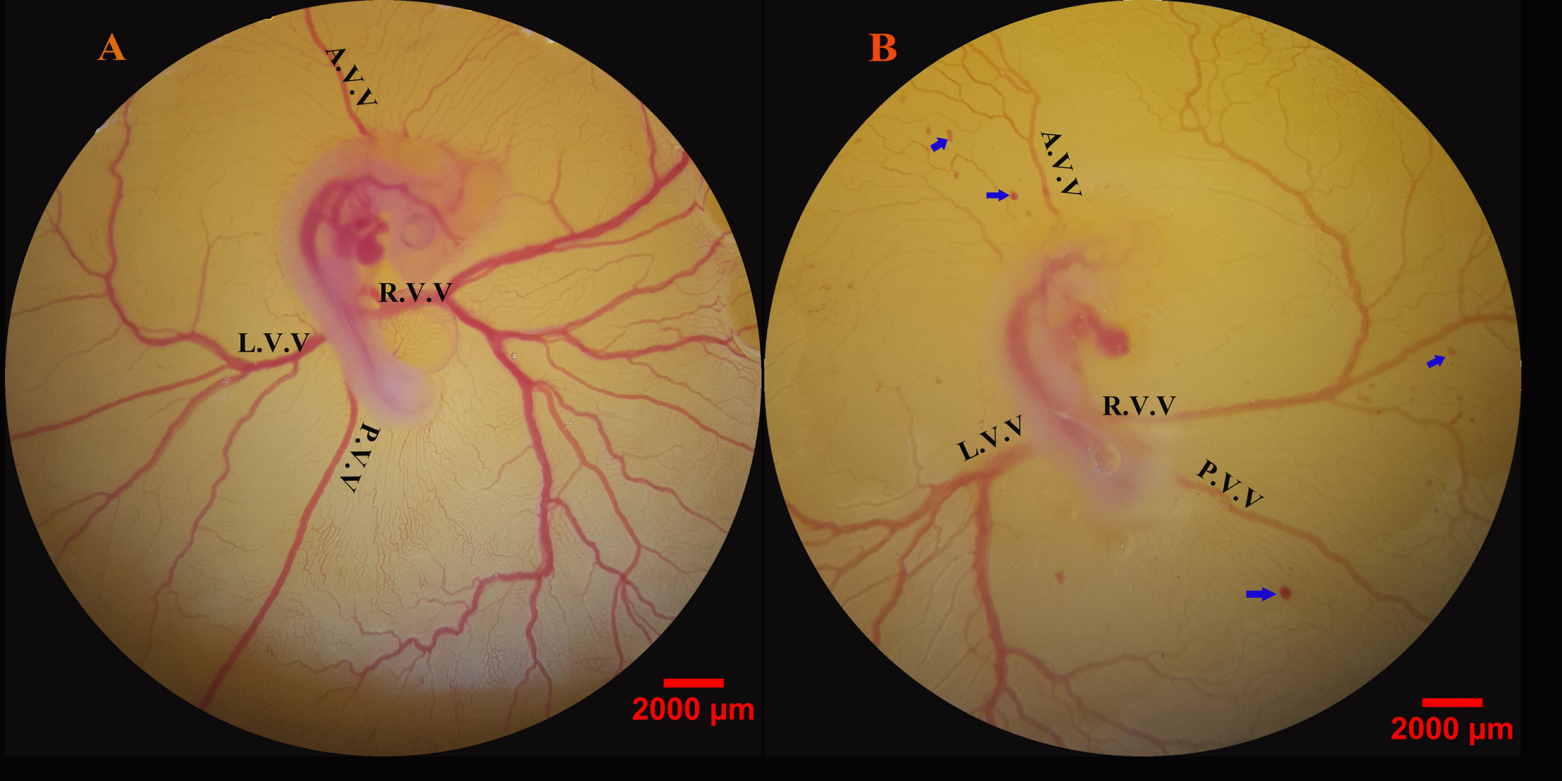
The chick’s extra-embryonic membrane vasculature at day 4 of the incubation period following meglumine antimoniate treatment. Embryonated eggs were treated three times at 24, 48 and 72 h of the incubation period. Control embryos with normal extra-embryonic membrane vasculature are seen (A). Embryonated eggs received meglumine antimoniate at the dosage of 150 mg per kg egg-weight (B). Vascular disruption is demonstrated by retarded branching and haemorrhages (blue arrows). A.V.V., anterior vitelline vein; L.V.V., left lateral vitelline vessel; P.V.V., posterior vitelline vein; R.V.V., right lateral vitelline vessel.

At the time of image acquisition (day 4 of incubation), the embryos were at Hamburger–Hamilton stages 22–24. In the control embryos, a rich network of vitelline circulation was around the embryo (Fig 6A). The blood, after circulating in the rich network of vessels, finally made its way either directly into vitelline veins, or to the sinus terminalis which acts as a collecting vessel, and then from the sinus terminalis to the vitelline veins. The vitelline veins converged towards the yolk-stalk and emptied into the omphalomesenteric veins. In the MA groups, a disturbing pattern of the EEM-vasculature was seen in the embryos, especially those treated with 150 mg per kg egg-weight. Vascular disruption was demonstrated by retarded branching or haemorrhages as depicted in Fig 6B.

**Fig 7:**
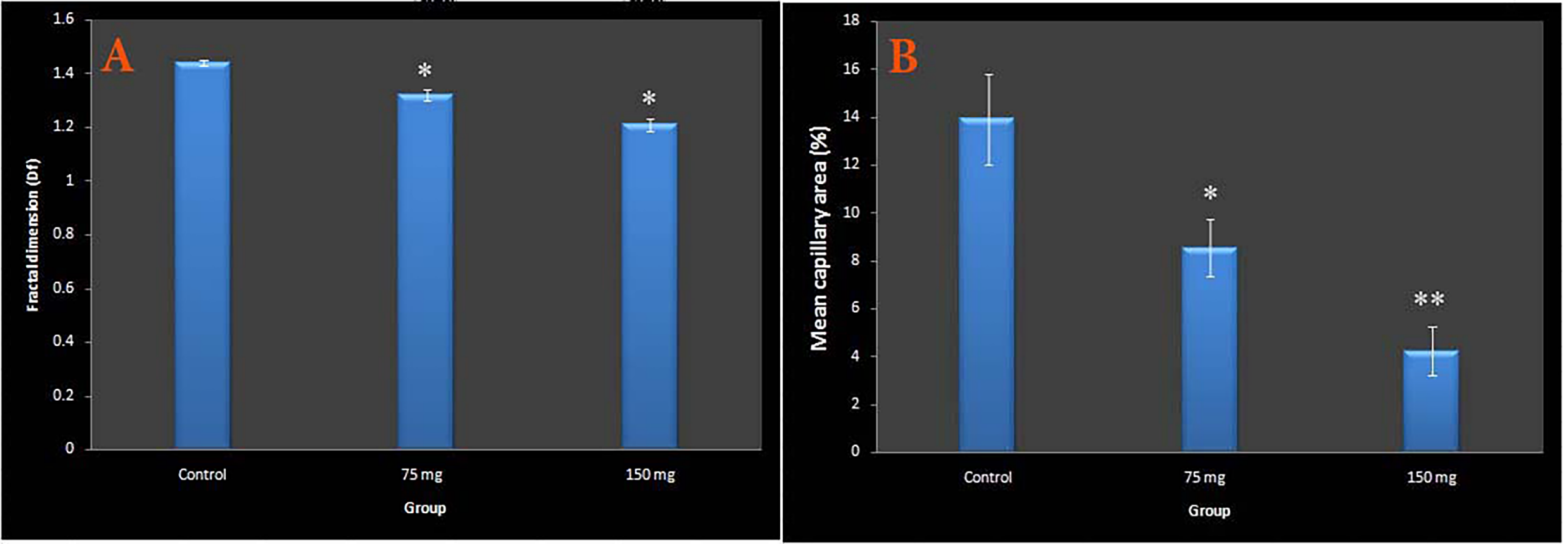
Effect of meglumine antimoniate on early embryonic vasculogenesis and angiogenesis using the fractal dimension (Df) and mean capillary area (MCA) quantification. Data are presented with comparison between the control (n = 10) and meglumine antimoniate at dosages of 75 (n = 10) or 150 (n = 10) mg per kg egg-weight. The Df was reduced in meglumine antimoniate-treated groups (A). The MCA was reduced in meglumine antimoniate-treated groups (B). Error bars exhibit standard error of mean; *p<0.05, **p<0.001, one-way ANOVA.

As mentioned previously, Df was used to analyze the vascular branching pattern. The Df value of the vessel plexus exposed to MA was reduced when compared with the controls (control, 1.44 ± 0.012 (95% CI 1.41–1.47); 75 mg per kg egg-weight, 1.32 ± 0.020 (95% CI 1.28–1.37); 150 mg per kg egg-weight, 1.21± 0.022 (95% CI 1.16–1.26); p<0.001) (Fig 7A). The reduction was associated with embryos that received the highest dose of the drug (p<0.001).

### Capillary density

**Fig 8:**
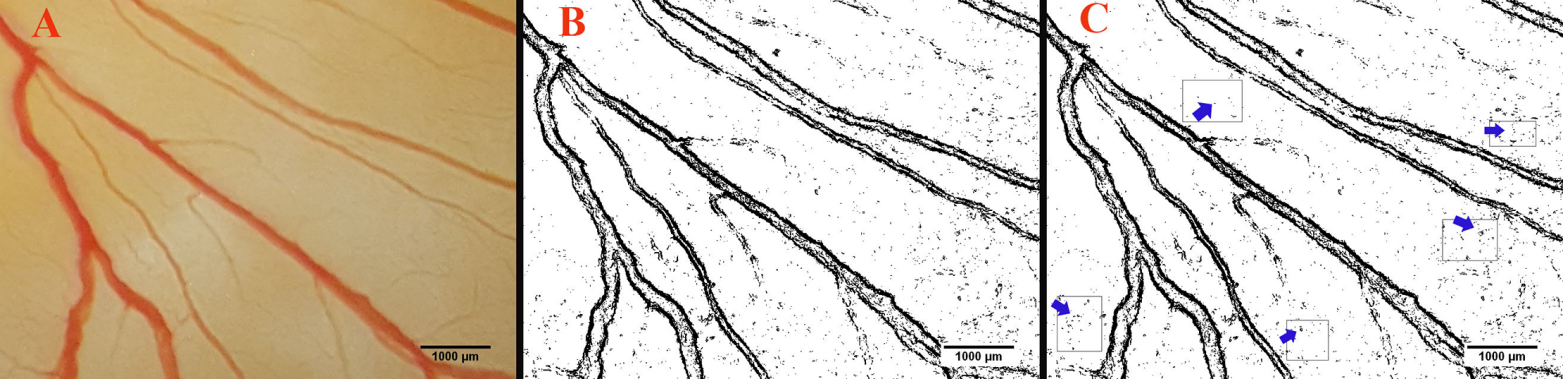
Quantify Quantification of the mean capillary area (MCA) from the captured images. The MCA quantified from the chick’s extra-embryonic membrane at day 4 of the incubation period. Selected area within the right-lateral vitelline vascular plexus (A). Selection has been was converted to a binarized image (B). Five areas (arrows) without any branch vessels are were selected and the percentage of the areas containing black pixels were was calculated for quantification of the MCA (C). The black pixels of the image indicate the red color, or blood, in the original main image.

The response to MA applied on the surface of the chick’s EEM at 24, 48 and 72 h of embryonic development is illustrated in Fig 7B. There was a considerable loss of MCA from the plexus of the treated embryos in both groups that received MA at dosages of 75 or 150 mg per kg egg-weight (control, 13.93 ± 1.89(95% CI 10.12–17.75); 75 mg per kg egg-weight, 8.54 ± 1. 20 (95% CI 6.13–10.96); 150 mg per kg egg-weight, 4.22 ± 1.01 (95% CI 2.19–6.25); p<0.05). Statistical analysis revealed that embryos that received high dosages of drug, exhibited a decrease in MCA in a dose-dependent manner (S4 Table). Quantify the mean capillary area (MCA) from the captured images is presented in Fig 8.

### VEGF-A and VEGF-R2 expression

**Fig 9:**
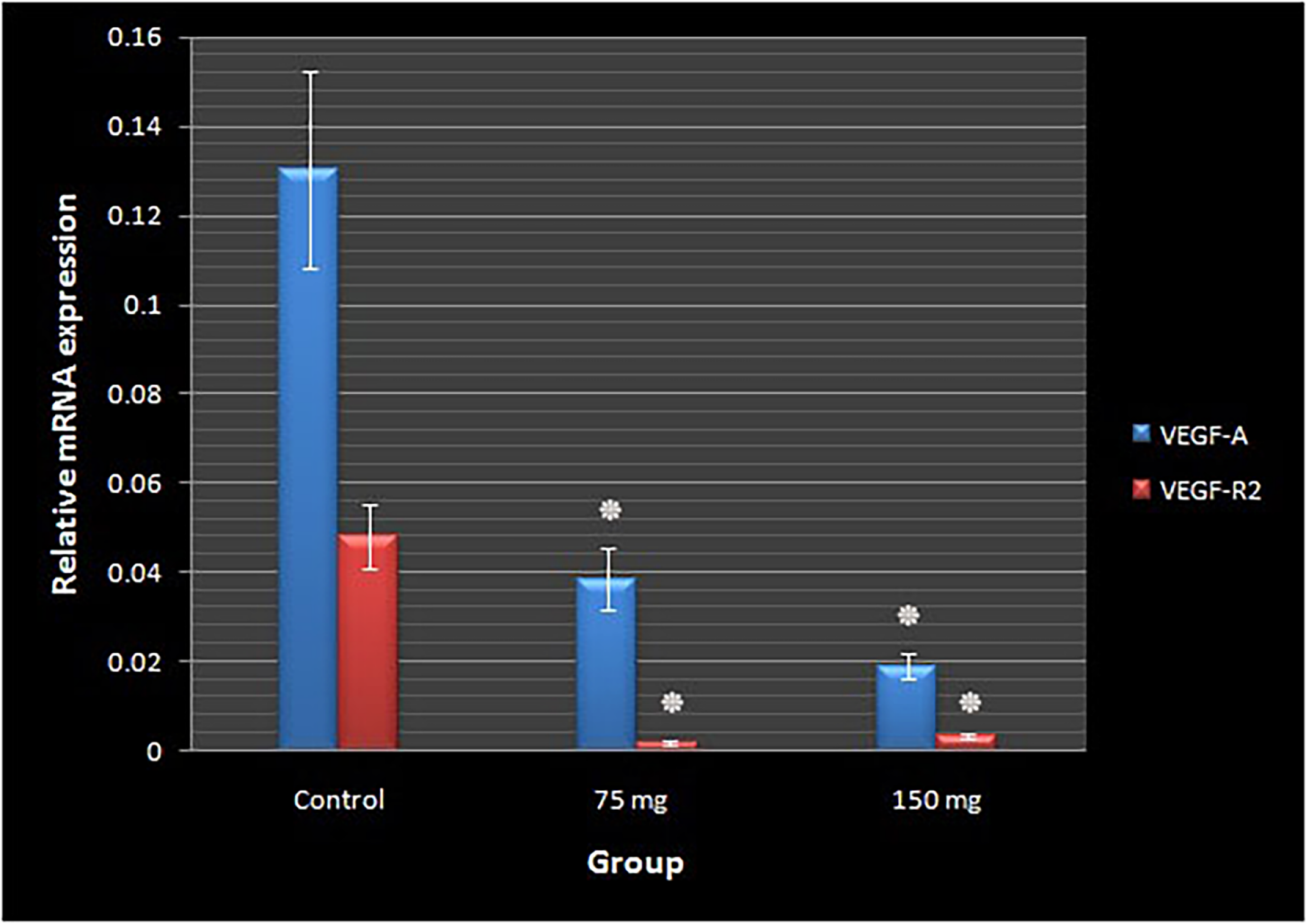
Relative mRNA expression levels of VEGF-A and VEGF-R2 genes following meglumine antimoniate treatment. The expression of VEGF-A and VEGF-R2 mRNA in the chick extra-embryonic membranes (n = 4 per experimental group) decreased in the meglumine antimoniate-treated groups as compared to the controls. Meglumine antimoniate administered at dosages of 75 or 150 mg per kg egg-weight (error bars show standard error of mean; *p<0.001, one-way ANOVA).

The expression of VEGF-A and VEGF-R2 genes was determined by qPCR assay at day 4 of the incubation period. The relative mRNA expression of VEGF-A and VEGF-R2 decreased in the MA-treated groups at dosages of 75 or 150 mg per kg egg-weight as compared to the control (Fig 9) (S1 File).

## Discussion

Data from the current study show that the major toxic effects of MA, at dosage of 75 mg per kg (the recommended human therapeutic dose), were at the second trimesters of the growing period. Severity of the gross and histopathological lesions was highest in the embryos that were treated with the highest dose of the drug. The most severe embryo-toxic effect produced by higher dosage of MA is also supported by some experimental data [4].

In this study, MA was injected into the yolk sac of embryonated eggs. It is shown that developmental toxicity, following administration of MA, is dependent on the length of treatment [4]. This phenomenon prompted the authors to enhance the drug resorption period so that in the present work, drug was injected into the yolk sac to cover the whole duration of embryo organogenesis (days 1–18). It should be noted that a successful treatment of leishmaniasis requires periods of 21–28 daily dosages of MA[22]. Gross lesion such as haemorrhages was also noted in the chick’s EEM and the lesion was less severe in the group that was treated with lower dose. Experimentally induced embryo-toxicity by MA has been reported in some species. For example, following subcutaneous injection of Wistar rats with MA, embryo-lethal and teratogenic effects such as atlas bone anomaly occurred spontaneously in the embryos [23]. Augmented soft-organs (e.g. dilated ureter) and skeletal injuries (misaligned sternebrae and rudimentary cervical ribs) were also reported [4]. In contrast, Santos et al. (2008) reported that MA can be administrated in the maternal organism without damaging the future offspring[24]. In some instances, the safe level of MA with no embryo-toxicity outcome in Wistar rats was 75 mg/(kg body-weight day) [4].

The pathogenesis, pharmacokinetics and tissue distribution of MA in embryo are not clearly defined. Based on the current results, it is concluded that MA can be distributed to the brain, kidney, heart and liver of the chick embryos, since the microscopic lesions were noticed in these organs. Other visceral abnormalities were not evaluated; therefore, demonstration of the histopathological lesions at the maintained tissues does not exclude the possibility that other organ anomalies might occur due to the exposure dosages. Hence, it would be useful to identify further details involved in the embryo-pathogenicity of MA in the future. The second indication of MA disorders was growth retardation and decreased body weight. There are some studies in the literatures, which focused on the embryo weight following treatment with pentavalent antimony compounds. Alkhawajah et al. (1996) treated 15 pregnant Sprague-Dawley rats with MA on day 6 and found that fetuses from treated mothers had a lower body weight[25]. The reduction of the fetal weight, following MA exposure, is also reported by others [26]. Similarly, in the current study, the embryos that received MA, especially at dose of 150 mg per kg egg-weigh, showed a significant weight loss.

The third indication of the MA disorders was considerable changes in some biochemical parameters of the chick’s amniotic fluid such as ALP, ALT, AST and amylase following MA treatment. Amniotic fluid has a very complex dynamic property surrounding the embryo, and monitoring it has wide usage in clinical diagnosis as an indicator of embryo status during the developmental stage. ALP is an enzyme found in some avian tissues, including bone, kidneys, intestine and liver. ALT is present in the cells of various avian tissues and its change is due to tissue injury. AST is the intracellular enzyme useful for detecting hepatocellular disruption and elevation is frequently associated with liver lesion. In birds, the pancreas, liver and small intestine produce amylase. Elevations have been associated with pancreatitis, enteritis and liver injury. In the current study, an increase in the above mentioned parameters of the amniotic fluid is suggested due to tissue injuries following MA treatment and possible transfer of the enzymes into amniotic fluid, since injuries in the brain, kidney, liver and heart were shown in the investigation. Alterations in the biochemical parameters of amniotic fluid due to teratogenicity agents have been reported earlier [19]. The present study shows that embryo-toxicity with alteration in the normal nature of the amniotic fluid can be generated by MA exposure during the growing period of the embryo.

The last indication of MA disorders is inhibition of the early vascular development in EEM and decrease in the expression of VEGF-A and its receptor. This altered vascular pattern may offer a link between MA exposure and developmental abnormalities, such as embryonic malformations, growth retardation and pathological lesions, which were observed in the current study. MA was used at dosages of 75 or 150 mg per kg egg-weight in the embryonated eggs. The former dosage is comparable to the routine therapeutic dosage that is used to treat leishmaniasis in human [22]. This dosage seems to have the tendency towards an adverse effect of MA on EEM-vasculature and gene expression. The pathogenesis and embryo-toxic effect of pentavalent antimony compounds on embryo are supported by some experimental data on drug behavior and pharmacokinetics. For example, the lesions may be due to the accumulation of drug in embryonic tissues. In humans, it was indicated that repeated dosage of MA caused an accumulation of drug in the body [23]. Toxicity of MA may also be due to osmolarity alteration [26].

The next explanation for the injuries of the affected tissues is the production of trivalent antimony compound. It was postulated by Chulay et al. (1988) that pentavalent antimony compounds can be converted to trivalent compounds during administration[27]. Trivalent compounds are generally more toxic than pentavalent ones and might be related to the toxic effect of the drug [28]. Furthermore, certain inherent characteristics of MA could be associated with its adverse effects. For example, some findings indicate that the drug has a geno-toxical property and acts as a pro-mutagenic compound that causes damage to DNA[3]. Moreover, MA is able to induce cell death by necrosis[29]. These activities of MA may also be involved in anti-vasculogenesis and anti-angiogenesis, which explain the reduction of the vascular plexus in injected embryos in the current investigation.

We have displayed that MA exposure affects the expression of VEGF and VEGFR-2. To explain the reduced expression of those genes, the following mechanisms were suggested: The cell damage, with successive decrease of vasculogenesis and angiogenesis, which is elicited by MA may have caused limited flow of blood via the vessels. This decreased blood flow correlated with some previous data that MA induces anemia and reduction in hemoglobin[30,31]. Alterations in blood flow may cause a reduction in shear stress, which is detected by the endothelial cells[32]. Generally, when shear stress increases, VEGF-A and VEGFR-2 are upregulated [33]. Therefore, a suggested reduction in shear stress after MA treatment may reduce VEGF-A and VEGFR-2 expressions. Furthermore, VEGF-A has different functions in vascular growth. For example, it stimulates VEGF-R2-mediated DNA synthesis[34]. Thus, reduction in VEGF-A will result in decreased VEGFR-2. This may explain the decrease in expression of VEGFR-2 in the vascular beds of MA-treated embryos.

In the present work, two methods that were used to explore the anti-vasculogenic/anti-angiogenic effect of MA are fractal analysis and calculation of the mean capillary area of the acquired images from the EEM-vasculature. So far, these methods have been widely applied in vascular studies[35,36]. The chick EEM-vasculature is fractal because it arranges within an almost two-dimensional plane and there is little crossing over of vessels[37]. The time of treatment was also selected to match with the previously recognized sensitive time to teratogenesis, in which vascular abnormalities had been documented [20,38]. To the best of the author’s knowledge, this is the first study which targeted different embryonic lesions following MA exposure, with the help of a chick embryo model. The study provides elaborate data on the toxico-pathological aspects of MA compounds. The findings are in line with previous limited reported data that focused on the toxicity of MA to the embryo. Additionally, this data show that MA not only has adverse effect on the embryo and biochemical parameters of the amniotic fluid, but also limits the early embryonic vasculogenesis/angiogenesis and results in devastating consequences.

## Conclusion

In conclusion, chick embryo is a preclinical model which is relevant for the assessment of adverse effects of drugs, whereby the interventions cannot be done in human fetus due to ethical reasons, but will inspire researchers and clinicians for specific changes in the design of subsequent drug prescription in patients. The results obtained in this study suggest that the use of pentavalent antimony compounds during pregnancy should be considered as potentially embryo-toxic, at least until further data are provided on safety for human fetus. Therefore, the drug prescription should be limited during pregnancy or only given when benefit outweighs risk, particularly in rural communities, because most of the infected patients live in remote areas which enhance the chance of contracting the disease and in turn, MA therapy. Furthermore, MA given to the chick embryo model was embryo-toxic at dosage equal to or higher than 75 mg/kg egg weight. The dosage found to be most severely embryo-toxic in the current study was 2 times higher than the recommended human therapeutic level. A lower dosage given in the early stage of pregnancy caused a far less pronounced embryonic lesion. Therefore, until the development of an effective prophylactic or therapeutic treatment modality, the use of safe alternatives should be of great priority. Since there are inadequate reports in this field, the results of this study can clarify some aspects of the pathogenesis caused by the side effects of MA during pregnancy.

## Supporting information

S1 Fig Gross lesions. Gross lesions of the meglumine antimoniate in the chick embryo model (TIF).
(RAR)

S2 Fig Histopathological lesions. Histopathological lesions of the meglumine antimoniate in the chick embryo model (TIF).
(RAR)

S1 Table Embryos weight. Date set for the weight of the chick embryos following meglumine antimoniate treatment. The weight is presented at days 6, 12 and 18 of the incubation period with comparison between control (n = 30) and meglumine antimoniate-treated embryos at dosages of 75 (n = 30) or 150 (n = 30) mg per kg egg-weight (XLSX).
(RAR)

S2 Table Biochemical parameters. Date set for biochemical parameters of amniotic fluid in chick embryo following meglumine antimoniate treatment. Data are acquired on the 14^th^ day of the incubation period. ALP, alkaline phosphatase; AST, aspartate aminotransferase; ALT, alanine aminotransferase; CT, creatinine (XLSX).
(RAR)

S3 Table Fractal dimension values. Date set for effect of meglumine antimoniate on the early embryonic vasculogenesis and angiogenesis using the fractal dimension (Df) quantification. Data are presented with comparison between control (n = 10) and meglumine antimoniate at dosages of 75 (n = 10) and 150 (n = 10) mg per kg egg-weight (XLSX).
(RAR)

S4 Table Mean capillary area values. Date set for effect of meglumine antimoniate on the early embryonic vasculogenesis and angiogenesis using the mean capillary area (MCA) quantification. Data are presented with comparison between control (n = 10) and meglumine antimoniate at dosages of 75 (n = 10) or 150 (n = 10) mg per kg egg-weight (XLSX).
(RAR)

S1 File Rotor-Gene data. Rotor-Gene experiment for determine the Relative mRNA expression levels of VEGF-A and VEGF-R2 genes following meglumine antimoniate treatment. The meglumine antimoniate was administered at dosages of 75 or 150 mg per kg egg-weight. vas: VEGF-A; kdr: VEGF-R2; gap: GAPDH, x: meglumine antimoniate at dosage of 75 mg per kg egg-weight; 2x: meglumine antimoniate at dosage of 150 mg per kg egg-weight; C: control (REX).
(RAR)
